# Preoperative risk stratification for patients with ≤ 1 cm papillary thyroid carcinomas based on preoperative blood inflammatory markers: construction of a dynamic predictive model

**DOI:** 10.3389/fendo.2023.1254124

**Published:** 2023-12-22

**Authors:** Lingqian Zhao, Tao Hu, Yuan Cai, Tianhan Zhou, Wenhao Zhang, Fan Wu, Yu Zhang, Dingcun Luo

**Affiliations:** ^1^ Zhejiang Chinese Medical University, Fourth Clinical Medical College, Hangzhou, Zhejiang, China; ^2^ Hangzhou First People’s Hospital, Department of Oncological Surgery, Hangzhou, Zhejiang, China; ^3^ Hangzhou Traditional Chinese Medicine Hospital Affiliated to Zhejiang Chinese Medical University, The Department of General Surgery, Hangzhou, Zhejiang, China; ^4^ The First Affiliated Hospital of Zhejiang Chinese Medicine University, Department of Urology1and Pathology2, Hangzhou, Zhejiang, China

**Keywords:** papillary thyroid carcinoma (PTC), dynamic predictive model, risk stratification, nomogram, neutrophil/lymphocyte ratio (NLR)

## Abstract

**Objective:**

The aim of this study was to investigate the relationships and predictive value of preoperative peripheral blood inflammatory markers as a means by which to assess risk for patients with ≤ 1 cm papillary thyroid carcinomas (PTCs). In addition, a preoperative risk stratification predictive model was constructed and validated.

**Methods:**

Clinical and pathologic data, as well as preoperative blood specimens, were collected from patients who underwent initial thyroid cancer surgery at the Hangzhou First People’s Hospital, from January 2014 to January 2023. Risk assessment was performed based on postoperative pathology according to the 2015 ATA guidelines for recurrence risk stratification. Using univariate analysis and multivariate logistic regression, we identified independent risk factors associated with risk stratification. A predictive model was established and its discriminative and calibration abilities were validated. An independent validation dataset was used to verify the model, and the model was deployed as an online calculator.

**Results:**

A total of 1326 patients were included in the study, with 1047 cases (79.0%) classified as low risk and 279 cases (21.0%) classified as intermediate to high risk. The modeling group consisted of 981 cases, through univariate analysis and multivariate logistic regression analysis, preoperative blood Neutrophil/Lymphocyte Ratio (NLR), gender, tumor diameter, and multifocality were identified as independent risk factors that distinguished between low and intermediate to high risk patients with ≤ 1 cm PTCs. The clinical predictive model exhibited an AUC of 0.785, specificity of 70.6%, and sensitivity of 75.8%. For the independent validation group of 345 patients, the AUC was 0.813, specificity was 83.8%, and sensitivity was 70.4%. The calibration curve and clinical decision curve indicate that the model demonstrates excellent calibration performance.

**Conclusion:**

A dynamic clinical predictive model based on preoperative blood NLR and clinical information for patients with ≤ 1 cm PTCs was established. The model is useful for preoperative risk assessment of patients with ≤ 1 cm PTCs.

## 1 Introduction

Thyroid cancer is one of the most common malignancies of the endocrine system. Among its various types, papillary thyroid carcinoma (PTC) is the most frequently encountered. With surgical intervention and hormonal suppression therapy, favorable prognoses can generally be achieved. PTC patients with a tumor diameter ≤ 1 cm are often asymptomatic when detected at an early stage. The majority of such cases are classified as low-risk following initial surgery. Therefore, in order to avoid unnecessary intervention, some experts propose that active surveillance (AS) of PTCs ≤ 1 cm is a suitable alternative to surgical treatment (1). However, within the ≤ 1 cm PTC category, 23.6%–31.1% of the carcinomas are identified as intermediate or high-risk (2, 3). Moreover, these cases are likely to have a poor initial treatment response, resulting in adverse outcomes such as lymph node recurrence and distant metastasis, with significant impact on patient survival (4). Thus, for patients with ≤ 1 cm PTCs, there exist varying degrees of risk. The ability to stratify risk levels among patients with ≤ 1 cm PTCs would greatly facilitate the formulation of treatment strategies. Currently, preoperative blood inflammatory markers, such as the Neutrophil/Lymphocyte Ratio (NLR), Lymphocyte/Monocyte Ratio (LMR), Platelet/Lymphocyte Ratio (PLR), and Systemic Immune-inflammation Index (SII) are widely used in the fields of oncology and inflammation. These markers have demonstrated promising efficacy in tumor prediction (5). The objective of this study was to explore the relationships among preoperative blood inflammatory markers and risk stratification (low, intermediate, and high) for patients with ≤ 1 cm PTCs, and as well as to determine the predictive value of these markers. A predictive model was established and validated.

## 2 Materials and methods

### 2.1 General information

Clinical and pathologic data of patients who underwent initial thyroid cancer surgery at the Hangzhou First People’s Hospital, were collected for analysis from January 2014 to July 2021. Patient data were used to establish a predictive model. Clinical and pathologic data of patients who underwent initial thyroid cancer surgery at the same hospital from August 2021 to January 2023 were collected as an independent validation group.

Inclusion criteria were as follows: ① Postoperative pathologic diagnosis of ≤ 1 cm PTC, ② Unilateral thyroid lobectomy with isthmusectomy or total thyroidectomy, along with prophylactic central compartment lymph node dissection and therapeutic lateral neck lymph node dissection.

Exclusion criteria were as follows: ① Presence of other malignant tumors or a history of neck radiation, ② Coexistence of hematological disorders, ③ Coexistence of autoimmune diseases, including Hashimoto’s thyroiditis, ④ Preoperative occurrence of acute or chronic inflammation or other diseases affecting blood routine examination results, ⑤ Incomplete preoperative clinical and pathologic data.

### 2.2 Data collection

Peripheral blood samples were collected from eligible patients within 1 week prior to thyroid cancer surgery. The samples were obtained in the morning after fasting, and a fully automated blood cell analyzer (Mindray BC-6800) was used for the analysis. The following data were manually collected: absolute lymphocyte count, absolute monocyte count, absolute neutrophil count, and absolute platelet count. Additionally, the Neutrophil/Lymphocyte Ratio (NLR), Platelet/Lymphocyte Ratio (PLR), Lymphocyte/Monocyte Ratio (LMR), and Systemic Immune-Inflammation Index (SII) were calculated and recorded.

General clinical information of the enrolled patients, including gender and age, were collected. Thyroid ultrasound images during preoperative thyroid cancer diagnosis were obtained, including tumor diameter, unilateral or bilateral involvement, and multifocality. Based on the final pathologic information, including cervical lymph node metastases, microscopic/macroscopic extrathyroidal extension, lymph node metastasis size, and distant metastasis, we applied the risk stratification criteria from the 2015 ATA guidelines to assess the preoperative risk stratification for ≤1cm PTC.

For lymph nodes exhibiting metastasis, the maximum diameter of the metastatic focus was measured. Two independent pathology experts performed microscopic individual measurements of the maximum diameter of the largest metastatic focus in the involved lymph node. In cases where there was disagreement regarding the diameter of the lymph node metastasis, a third pathology expert was invited to provide a final determination. Descriptive statistics were used to present quantitative data as means ± standard deviation ( $\bar{X}$ ± S), while frequency and percentages were used for categorical data.

### 2.3 Statistical methods

Statistical analysis of the modeling group data was performed using SPSS software (version 26). Independent sample T-tests were used for comparing quantitative data between two independent groups, while chi-square tests were employed for comparing categorical data between groups. Based on the risk statistical differences, risk factors related to preoperative blood inflammatory markers, clinical information, and pathologic characteristics were identified. Analysis of the correlation between preoperative blood indicators and cervical lymph node metastasis, including central lymph node metastasis (CLNM) and lateral lymph node metastasis (LLNM), with PTC ≤ 1cm. Conducting collinearity analysis to assess inter-variable correlations and eliminate confounding factors. Logistic regression analysis was conducted for selected risk factors to determine those factors most associated with risk stratification. Odds ratios (OR) and their 95% confidence intervals (95% CI) were calculated to identify independent risk factors influencing risk stratification for patients with ≤ 1 cm PTCs.

Using R Studio (version 1.4) and relevant packages, a clinical prediction model was established based on the identified independent risk factors. Logistic regression was applied to the modeling group data to construct a clinical prediction model. Receiver Operating Characteristic (ROC) curve analysis, Area Under the Curve (AUC) values with their 95% confidence intervals (95% CI), Youden index, sensitivity, and specificity were used to evaluate the predictive performance and discriminatory ability of the model.

To internally validate the established clinical prediction model, Bootstrap resampling was performed 1000 times, and a calibration plot was generated to assess the consistency between the model’s predicted probabilities and the actual outcomes. Decision Curve Analysis (DCA) was conducted to evaluate the clinical net benefit of the model in the modeling group, considering the area between the curve and the diagonals representing full intervention (slanting line) and no intervention (horizontal line), as well as the threshold range.

The established model was independently validated using the validation group data. ROC analysis, AUC, 95% CI, Youden index, sensitivity, and specificity were employed to assess the predictive performance and discriminatory ability of the model in the independent validation group. DCA curves were utilized to evaluate the clinical net benefit of the model in the validation group. These approaches were used to evaluate the discriminatory ability and calibration of the clinical prediction model in an independent dataset. Based on the establishment and validation of the clinical prediction model, an online calculator was developed using the R language. P < 0.05 was considered statistically significant.

## 3 Results

### 3.1 General information

Based on the inclusion and exclusion criteria, 1326 patients with ≤ 1 cm PTCs were included in the study. Of these 1047 cases (79.0%) were classified as low risk and 279 cases (21.0%) were classified as intermediate-high risk. The modeling group consisted of 981 cases, with 770 cases (78.5%) classified as low risk and 211 cases (21.5%) classified as intermediate-high risk. Among these, there were 222 male patients (22.6%) and 759 female patients (77.4%). The validation group was comprised of 345 cases, with 277 cases (80.3%) classified as low risk and 68 cases (19.7%) classified as intermediate-high risk. Among these, there were 83 male patients (24.1%) and 262 female patients (75.9%). Detailed information regarding patients’ general characteristics and pathologic data are found in **Table 1**.

**Table 1:** General characteristics of the 1,326 patients in the modeling and validation groups.

		Modelling group (981)		Validation group (345)	
		Low risk	Intermediate-high Risk	Low risk	Intermediate-high Risk
Total		770	211	277	68
Sex	Male	164	58	56	27
	Female	606	153	221	41
Age	$\bar{X}$ ± S	46.6 ± 11.6	45.6 ± 13.3	44.7 ± 12.0	43.0 ± 12.2
Tumor size	$\bar{X}$ ± S	5.52 ± 2.05	7.72 ± 2.05	5.04 ± 2.01	7.53 ± 2.16
Multifocality	Yes	186	84	59	30
	No	584	127	218	38
Bilateral	Yes	128	63	41	22
	No	642	148	236	46
NLR	$\bar{X}$ ± S	1.82 ± 0.76	2.10 ± 1.05	1.81 ± 0.82	1.85 ± 0.76
PLR	$\bar{X}$ ± S	130.12 ± 44.18	130.62 ± 47.71	128.13 ± 38.56	132.21 ± 42.42
LMR	$\bar{X}$ ± S	6.12 ± 2.49	5.73 ± 2.28	5.29 ± 2.03	5.71 ± 2.23
SII	$\bar{X}$ ± S	410.05 ± 205.03	456.71 ± 228.39	411.33 ± 218.78	429.50 ± 210.01
CLNM	Yes	212	59	44	46
	No	558	152	233	22
LLNM	Yes	735	85	4	20
	No	35	126	273	48
TNM	I	710	164	267	60
	II	59	46	10	7
	III	1	1	0	1
	IV	0	0	0	0

**Figure 1:**
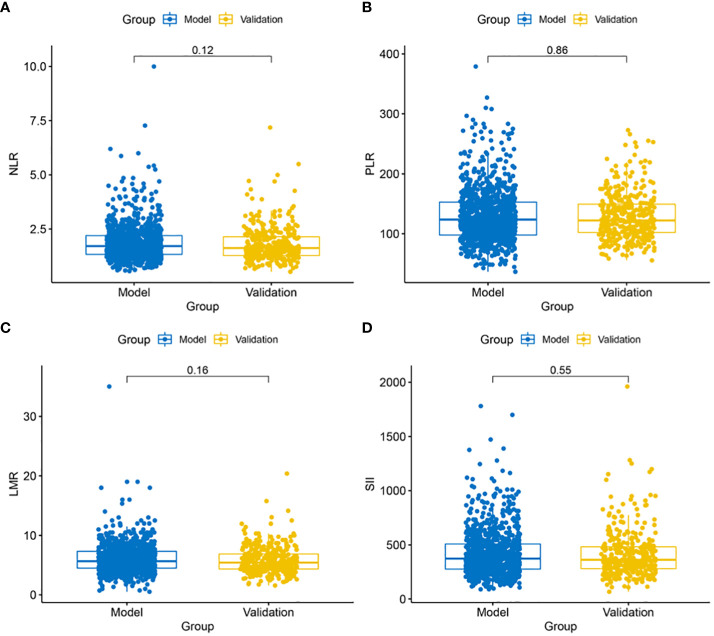
Comparison of blood marker levels between the modeling and validation groups. **(A)** No significant difference in NLR levels (P = 0.12) between the modeling and validation groups; **(B)** No significant difference in PLR levels (P = 0.88) between the modeling and validation groups; **(C)** No significant difference in LMR levels (P = 0.16) between the modeling and validation groups; **(D)** No significant difference in SII levels (P = 0.55) between the modeling and validation groups.

A comparison of preoperative blood data between the modeling group and the validation group revealed no significant difference in preoperative blood PLR, NLR, LMR, and SII levels, indicating no significant baseline differences in preoperative blood markers between the modeling and validation groups (**Figure 1**).

Through collinearity diagnosis, it was observed that the Variance Inflation Factors (VIF) for age (VIF=1.0), gender (VIF=1.1), blood NLR levels (VIF=2.2), blood LMR levels (VIF=1.3), blood PLR levels (VIF=2.1), and blood SII levels (VIF=5.0) were all below 10, indicating that NLR, LMR, PLR, and SII are not influenced by age or gender.

### 3.2 Screening of predictive model variables

T-tests and chi-square tests identified significant associations (P < 0.05) between preoperative blood NLR, LMR, SII levels, gender, tumor diameter, multifocality, bilateral tumors, and risk for stratification of patients with ≤ 1 cm PTCs. Age and preoperative blood PLR level were not significantly related to low or intermediate-high risk stratification of patients with ≤ 1 cm PTCs (**Table 2**).

**Table 2:** General characteristics and univariate logistic regression analysis of the 981 patients in the modeling group.

		Number of ≤ 1 cm PTC (%)	P-value (univariate analysis)	P-value (Logistic regression)	OR (Logistic regression)	95%CI (Logistic regression)
Total		981				
Gender	Male	222 (22.6%)	0.037	0.010	1.674	1.130–2.479
	Female	759 (77.4%)				
Age	$\bar{X}$ ± S	46.3 ± 12.0	0.308			
Tumor diameter	$\bar{X}$ ± S	5.99 ± 2.24	<0.001	<0.001	1.601	1.473–1.741
Multifocality	Yes	270 (27.5%)	<0.001	0.018	1.551	1.078–2.231
	No	711 (72.5%)				
Bilateral	Yes	191 (19.5%)	<0.001	0.362		
	No	790 (80.5%)				
NLR	$\bar{X}$ ± S	1.88 ± 0.84	0.001	0.002	1.360	1.119–1.653
PLR	$\bar{X}$ ± S	130.22 ± 44.93	0.886			
LMR	$\bar{X}$ ± S	6.03 ± 2.45	0.034	0.707		
SII	$\bar{X}$ ± S	420.05 ± 211.00	0.006	0.468		

The application of T-tests revealed that preoperative blood NLR, LMR, and SII levels exhibited no significant correlation with CLNM and LLNM in **Table 3** (*P* > 0.05).

**Table 3:** Analysis of the correlation between ≤1cm PTC and cervical lymph node metastasis with blood inflammatory indices.

		CLNM		P-value
		Yes	No	
NLR	$\bar{X}$ ± S	1.87 ± 0.81	1.88 ± 0.87	0.958
PLR	$\bar{X}$ ± S	127.48 ± 43.49	131.84 ± 45.72	0.143
LMR	$\bar{X}$ ± S	5.93 ± 2.39	6.10 ± 2.50	0.272
SII	$\bar{X}$ ± S	421.48 ± 198.74	419.21 ± 218.05	0.821
		LLNM		P-value
		Yes	No	
NLR	$\bar{X}$ ± S	1.91 ± 0.82	1.88 ± 0.84	0.712
PLR	$\bar{X}$ ± S	128.09 ± 39.29	130.52 ± 45.68	0.579
LMR	$\bar{X}$ ± S	5.70 ± 1.98	6.09 ± 2.51	0.106
SII	$\bar{X}$ ± S	429.84 ± 196.15	418.68 ± 213.06	0.601

Further, multivariate logistic regression analysis revealed that preoperative blood NLR level (*P* = 0.002, OR: 1.360, 95% CI: 1.119–1.653), male gender (*P* = 0.010, OR: 1.674, 95% CI: 1.130–2.479), tumor diameter (*P* < 0.001, OR: 1.601, 95% CI: 1.473–1.741), and multifocality (*P* = 0.018, OR: 1.551, 95% CI: 1.078–2.231) were independent risk factors for low and intermediate-high risk patients with ≤ 1 cm PTCs.

### 3.3 Establish a model and initially test the model

**Figure 2:**
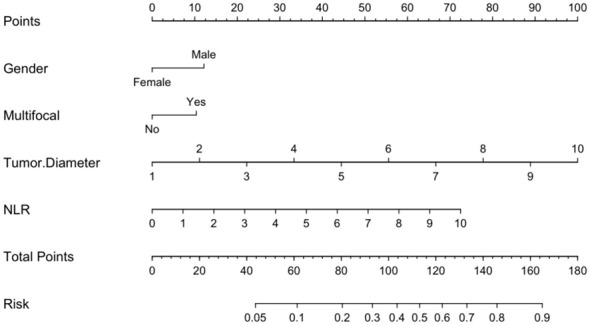
Preoperative prediction model for low-risk patients with ≤ 1 cm PTCs.

**Figure 3:**
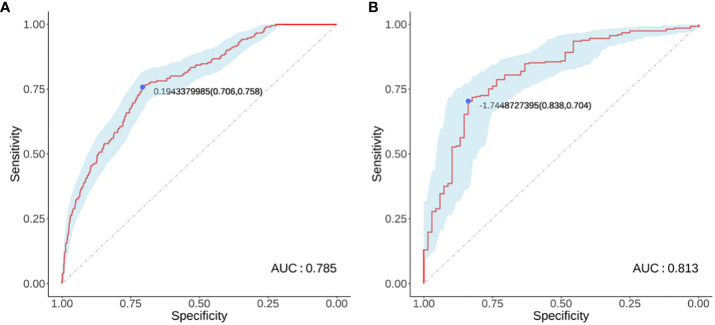
Modeling group **(A)** and Validation group **(B)** receiver operator characteristic curves. **(A)** ROC curve for the modeling group; **(B)** ROC curve for the validation group.

**Figure 4:**
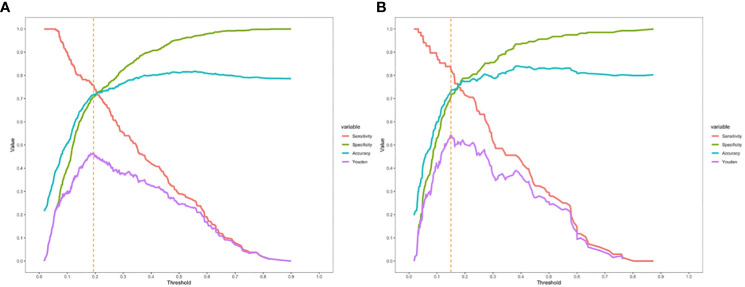
Evaluation curve of the predictive efficacy of clinical prediction model. **(A)** Evaluation curve of the predictive efficacy of clinical prediction model for the modeling group; **(B)** Evaluation curve of the predictive efficacy of clinical prediction model for the validation group.

Logistic regression analysis identified independent risk factors including; preoperative blood NLR levels, gender, tumor diameter, and multifocality. These independent predictive factors were combined to establish a clinical prediction model for distinguishing low-risk and intermediate-high-risk categories of patients with ≤ 1 cm PTCs (**Figure 2**). Logistic regression was used to construct an ROC curve, which revealed that the predictive model incorporating the aforementioned risk factors, namely preoperative blood NLR levels, gender, tumor diameter, and multifocality, had an AUC of 0.785 (95% CI: 0.751–0.819), predicting a low-risk for patients with ≤ 1 cm PTCs (**Figure 3A**). The evaluation curve of the predictive efficacy of clinical prediction model for modeling group demonstrated that the model had good discriminative ability with a specificity of 70.6%, sensitivity of 75.8%, and accuracy of 71.7% at the optimal Youden index (**Figure 4A**). When the Youden index is maximum, the cut-off value of NLR is 2.51.

**Figure 5:**
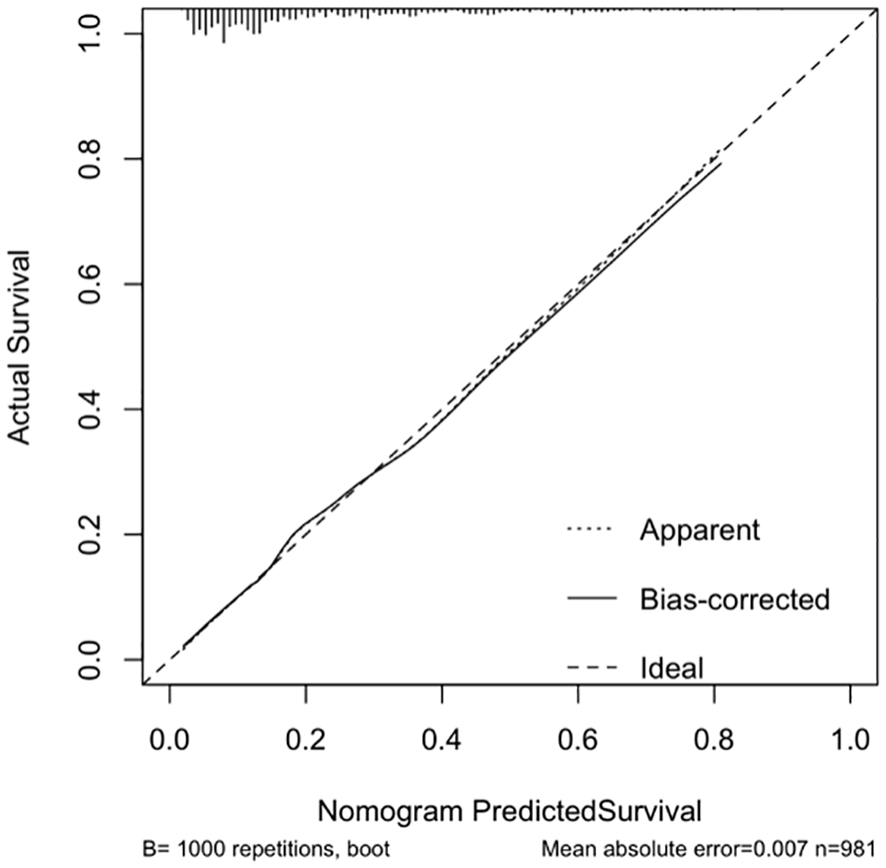
Modeling group calibration curve.

**Figure 6:**
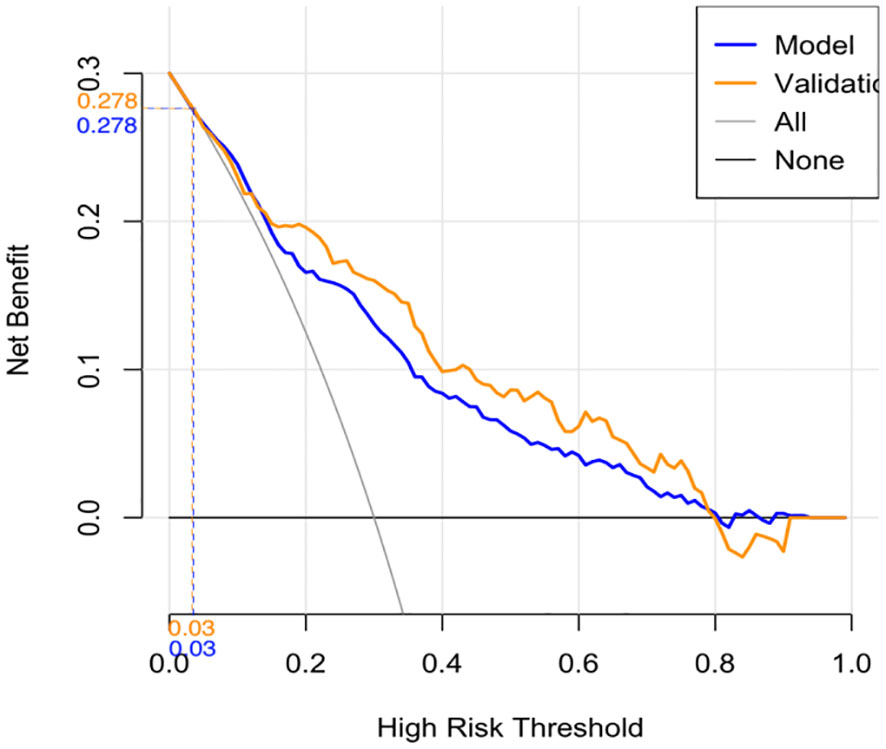
Modeling group and validation group decision curve analysis (DCA).

**Figure 7:**
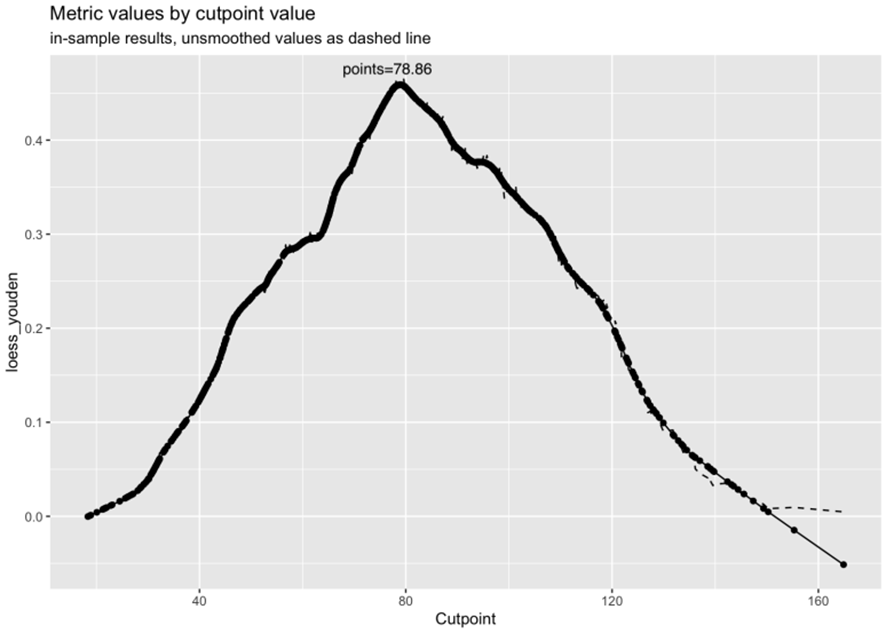
Youden’s curve and optimal cutoff value for predicting low-risk patients with ≤ 1 cm PTCs.

The calibration curve indicated a close correspondence between the predicted and actual values of the model, displaying good calibration with an average absolute error of 0.007 between the predicted and actual differentiation rate for low-risk and intermediate-high-risk patients with ≤ 1 cm PTCs (**Figure 5**). Decision curve analysis (DCA) showed that intervention within a threshold range of 0–0.8 resulted in greater net benefit compared to no intervention or universal intervention, indicating a degree of clinical utility for the model (**Figure 6**). Based on the model’s predicted values, the optimal cutoff value for predicting low-risk patients with ≤ 1 cm PTCs was calculated as 78.86 using the Youden index from the model’s ROC curve (**Figure 7**).

### 3.4 Independent model validation

Validation of the risk stratification predictive model for patients with ≤ 1 cm PTCs was performed with the validation dataset. Results found, at the maximum Youden index, an AUC of 0.813 (95% CI: 0.756–0.870) (**Figure 3B**). Evaluation curve of the predictive efficacy of clinical prediction model for the validation dataset found a specificity of 83.8%, sensitivity of 70.4%, and accuracy of 73.0% at the optimal Youden index (**Figure 4B**). These findings suggest that the model retains a good predictive discriminative ability with external independent data. Based on results using the validation dataset, DCA found intervention within a threshold range of 0–0.8 yielded significantly greater net benefit compared to no intervention or universal intervention, indicating that the model had clinical utility for the validation dataset (**Figure 6**).

### 3.5 Establishment of a web-based predictive model

**Figure 8:**
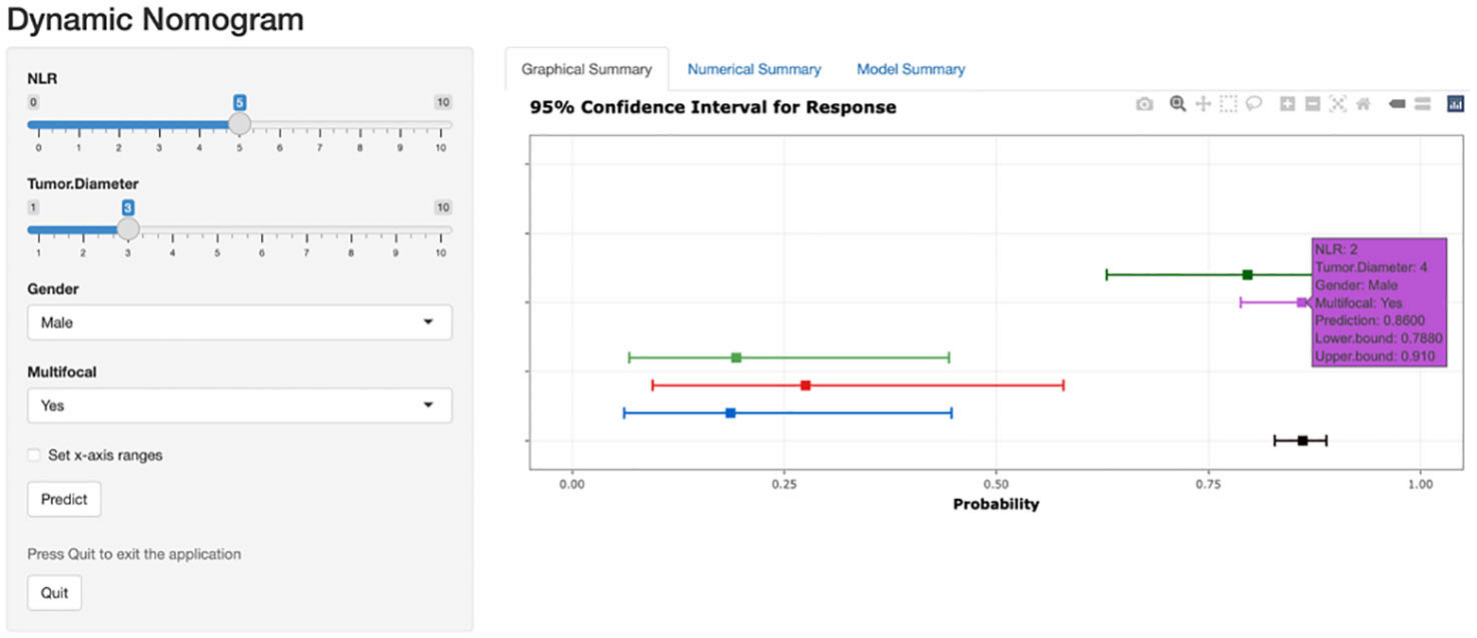
Web-based dynamic prediction model for preoperative prediction of low-risk patients with ≤ 1 cm PTCs. (https://zzz030.shinyapps.io/DynNomapp/).

Based on the risk stratification clinical predictive model for patients with ≤ 1 cm PTCs, a web-based calculator was developed in the R programming environment and deployed on a website (https://zzz030.shinyapps.io/DynNomapp/). Upon entering patients’ clinical information, the online calculator automatically computes risk stratification. Through simulation testing with multiple datasets, the web application demonstrated stable performance, indicating the effectiveness of the dynamic predictive model (**Figure 8**).

## 4 Discussion

Predicting the risk stratification of PTC with a diameter ≤1cm prior to surgery would greatly facilitate the development of treatment plans for such cases, benefiting a larger number of patients. Some experts also propose that for ≤1cm PTC tumors with low predicted invasiveness or risk, it might be possible to consider less extensive surgical interventions or even adopt active surveillance (6), while cases with higher predicted risk are recommended to undergo thorough preoperative examinations, surgical treatments, and postoperative follow-up (7).

However, several challenges exist for preoperative risk stratification of patients with ≤ 1 cm PTCs. First, there is no unified treatment standard for such patients. Currently, South Korea (1) and Japan (8) are conducting prospective active surveillance clinical studies for low-risk PTC patients with a tumor diameter ≤ 1 cm. Those studies define low-risk cases as those without extrathyroidal invasion, cervical lymph node metastasis, or distant metastasis. Although these indicators have been validated to impact PTC tumor progression, their direct relationship to tumor risk and their applicability to other geographical regions and institutions still require further investigation. Second, prediction models often rely on postoperative pathologic data. Wang (9) established a prediction model based on the postoperative pathology of 8,668 patients with ≤ 1 cm PTCs, finding relationships among extrathyroidal invasion and lateral neck lymph node metastasis and risk. However, clinical practitioners cannot foresee postoperative pathology, limiting the usefulness of such approaches. Third, predicting small-volume lymph node metastasis is challenging. The 2015 ATA guidelines established risk stratification criteria for postoperative pathological recurrence of PTC, including low recurrence risk assessment criteria such as no invasion of surrounding tissues, no distant metastasis, and a maximum of ≤ 5 metastatic lymph nodes with each lymph node metastasis measuring < 0.2 cm in diameter (referred to as small-volume lymph node metastasis). Previous studies have found associations among the number and maximum diameter of lymph node metastases and the malignancy of the primary tumor, as well as with postoperative local recurrence and survival prognosis for patients with PTC (10). Patients with small-volume or no lymph node metastasis demonstrate similar postoperative recurrence rates (11), which is significantly better than patients with meddle or large-volume lymph node metastasis (>5 lymph nodes and/or a metastatic focus diameter ≥ 0.2 cm) (12, 13). Therefore, PTC patients with small-volume lymph node metastasis may be defined as low-risk preoperatively, allowing for the selection of more suitable treatment approaches. However, assessment and prediction of PTC small-volume lymph node metastasis remain difficult.

The risk stratification criteria proposed by the 2015 ATA guidelines serves as a relatively accurate and reliable method for evaluation of the potential recurrence of thyroid cancer and to some extent reflect the invasiveness of the primary tumor (14). However, these criteria also have limitations, as they are based on postoperative pathologic information and cannot be used for preoperative prediction. Therefore, in this study, postoperative pathologic data were meticulously collected as the gold standard, including the number of lymph node metastases and the diameter of metastatic foci. Referring the recurrence risk stratification criteria of the 2015 ATA guidelines and incorporating multiple preoperative clinical factors, preoperative risk stratification for patients with ≤ 1 cm PTCs were predicted.

In addition to inflammatory and immune cell infiltration of tumor tissue, which can alter the tumor microenvironment and impact tumor occurrence and progression, inflammation-related markers in the blood also have an integral relationship with tumor development (15, 16). For patients with PTCs, multiple studies have demonstrated relationships among blood inflammatory markers and PTC risk (5, 17). Ceylan’s (18) investigation of blood levels of NLR and PLR revealed associations with the clinical and pathologic invasiveness of PTC. These results suggest that each of these potential biomarkers are useful for clinical risk assessment of PTC patients. Lang (19) identified a relationship between blood NLR levels and poor tumor characteristics. Korean scholar Kim (20) conducted a follow-up study of 542 PTC patients for 6 to 99 months and performed survival analysis, finding significant differences in blood NLR levels between stage III/IV and stage I/II PTC patients. Associations were also found between blood NLR levels and 5-year disease-free survival (DFS), indicating that blood NLR levels can serve as predictive markers for PTC risk. In contrast to other studies that stratified NLR values, this study conducted an analysis using continuous NLR values to reduce data bias. Results confirmed that blood NLR levels are independent risk factors for distinguishing low-risk and intermediate-to-high-risk patients with ≤ 1 cm PTCs (P < 0.05).

In this study, an increase in tumor diameter was accompanied by an increased risk, suggesting that larger tumor diameter should be considered during preoperative evaluation as well as tumor volume and tumor burden. Kuo et al. (21) found a higher incidence of tumor aggressiveness for multifocal PTCa compared to unifocal tumors. Feng (22) confirmed the association between multifocal PTC and tumor aggressiveness. In this study, multifocality was identified as an independent risk factor for intermediate high-risk patients with ≤ 1 cm PTCs.

The aim of this study was to differentiate between low-risk and intermediate-to-high-risk patients with ≤ 1 cm PTCs before surgery, rather than low-to-intermediate risk and high risk. It is important to note that some clinicians believe that the presentation of intermediate risk patients is similar to that of low risk patients (2). However, 14%–34% of intermediate-risk patients experience a biochemical or a structurally incomplete response after treatment (23). Previous research has been notably limited in its focus on risk stratification studies. The majority of these studies have exhibited a bias towards identifying risk factors for high-risk tumors, leaving a relative lack of emphasis on the identification of low-risk tumors. This approach resulted in overtreatment of low-risk tumors and insufficient treatment of intermediate high-risk tumors.

This study not only focused on risk assessment for patients with ≤ 1 cm PTCs but also emphasized the preoperative evaluation period. Previous studies of PTC risk stratification relied on postoperative histopathological data, primarily for developing postoperative follow-up plans and monitoring tumor recurrence. Furthermore, compared to more complex, costly, and non-standardized genetic testing methods, preoperative blood markers are accurate and easily accessible results. As demonstrated in clinical research conducted by the Muge Bilge team, blood NLR and PLR levels were found to be higher in Hashimoto’s thyroiditis patients with normal thyroid function compared to the healthy control group. This suggests that the influence of Hashimoto’s thyroiditis on blood inflammatory indices could potentially introduce confounding factors in this study, affecting model accuracy. Therefore, Hashimoto’s thyroiditis was not included in this study. Further, considering the relative difficulty of preoperative ultrasound assessment of capsular invasion and extrathyroidal extension in patients with ≤ 1 cm PTCs, this study did not include such data. The data evaluated in this study were accurate and clinically relevant information that can be obtained preoperatively, enabling a clear determination of patient treatment direction, facilitating model generalization. This study established a columnar chart for preoperative risk stratification for patients with ≤ 1 cm PTCs and plotted the evaluation curve of the predictive efficacy of clinical prediction model based on the predictive efficacy of the modeling and validation groups, clearly and intuitively reflecting the changes in sensitivity, specificity, and accuracy at different thresholds. Furthermore, this study utilized a web-based calculator to establish a dynamic prediction model, which aligns with the current digital working environment, making it more convenient and accessible compared to previous static prediction models. As such, the model is better suited to clinical practice. Simultaneously, it fosters enhanced clinical guidance, facilitating the selection of more appropriate preoperative assessments and surgical approaches, as well as instrumentation (24). This, in turn, is instrumental in mitigating the incidence of postoperative complications, underscoring a pivotal role in optimizing patient outcomes within a surgical context (25).

This study has several limitations: 1. The data used in this study came from a single research center, and future studies should include multicenter data for validation. 2. This study included four preoperative blood markers, and future studies should explore additional relevant markers related to patients with ≤ 1 cm PTCs. 3. The preoperative ultrasound indicators included tumor diameter, multifocality, and unilateral, or bilateral involvement. With the advancement of ultrasound technology, precise evaluation of other ultrasound indicators, such as extrathyroidal extension, would contribute to model development and application.4. In this study, LMR, PLR, and SII exhibited correlations with intermediate-to-high-risk categories within ≤1cm PTC during univariate analysis; however, they did not emerge as independent risk factors for recurrence in ≤1cm PTC. This phenomenon could be attributed to their potential indirect associations with low-risk and intermediate-to-high-risk subgroups of ≤1cm PTC, or it may be influenced by sample size constraints, necessitating further research for confirmation.

## 5 Conclusion

A dynamic clinical predictive model based on preoperative blood NLR and clinical information for patients with ≤ 1 cm PTCs was established. The model is useful for preoperative risk assessment of patients with ≤ 1 cm PTCs.

## Data availability statement

The raw data supporting the conclusions of this article will be made available by the authors, without undue reservation.

## Ethics statement

The studies involving humans were approved by Ethics Committee of Hangzhou First People’s Hospital. The studies were conducted in accordance with the local legislation and institutional requirements. The participants provided their written informed consent to participate in this study.

## Author contributions

LZ: Writing – original draft, Writing – review & editing. YC: Writing – original draft, Writing – review & editing. TZ: Writing – review & editing. WZ: Writing – review & editing. FW: Writing – original draft. TH: Writing – original draft. YZ: Writing – review & editing. DL: Funding acquisition, Writing – review & editing.
